# Диабетическая гастроэнтеропатия: современные методы диагностики и лечения

**DOI:** 10.14341/probl13082

**Published:** 2022-07-13

**Authors:** К. О. Кузнецов, А. Ю. Михеева, А. А. Ишмухаметова, Т. А. Толстых, А. Р. Галляметдинова, З. У. Ботирова, А. А. Забирова, А. Ш. Шарипова, А. Б. Шайхлисламова, Д. Р. Абрахманова

**Affiliations:** Российский национальный исследовательский медицинский университет им. Н.И. Пирогова; Национальный медицинский исследовательский центр им. В.А. Алмазова; Первый Московский государственный медицинский университет им. И.М. Сеченова; Первый Московский государственный медицинский университет им. И.М. Сеченова; Башкирский государственный медицинский университет; Башкирский государственный медицинский университет; Башкирский государственный медицинский университет; Башкирский государственный медицинский университет; Башкирский государственный медицинский университет; Башкирский государственный медицинский университет

**Keywords:** сахарный диабет, диабетическая гастроэнтеропатия, гастропарез, диарея, запор

## Abstract

Сахарный диабет является хроническим заболеванием с растущей распространенностью во всем мире, вместе с тем увеличивается и распространенность его осложнений, включая гастроэнтеропатию. Патофизиология диабетической гастроэнтеропатии (ДГ) объединяет гипергликемию, дисфункцию блуждающего нерва, снижение экспрессии синтазы оксида азота в миентеральном сплетении, изменения в интерстициальной клеточной сети Кахаля, а также окислительный стресс. Клиническими признаками ДГ являются гастроэзофагеальный рефлюкс, гастропарез, запор, боль в животе и диарея. Среди методов диагностики выделяют манометрию с измерением pH (оценка моторики пищевода), сцинтиграфию опорожнения желудка, дыхательный тест (с целью оценки гастропареза), аспирацию и культивирование содержимого тощей кишки (с целью диагностики синдрома избыточного бактериального роста). На сегодняшний день не существует окончательного лечения ДГ — междисциплинарный подход направлен на замедление прогрессирования заболевания, облегчение симптомов и восстановление функции желудочно-кишечного тракта. Пациентам рекомендуются диета с низким содержанием простых сахаров и высоким содержанием клетчатки, оптимизация гликемического контроля при целевой гликемии менее 180 мг/дл. Что касается медикаментозной терапии, оправдано использование прокинетиков и противорвотных средств, а при возникновении синдрома избыточного бактериального роста — проведение антибактериальной терапии (рифаксимин). Также накапливаются современные подходы к лечению ДГ, включая использование ботулинического токсина, пилоропластику и электрическую стимуляцию желудка у отдельных пациентов. Несмотря на постоянную разработку новых методов лечения, они пока не способны полностью излечить ДГ в ближайшем будущем, что делает необходимым проведение дальнейших исследований в данной области.

## Abstract (en)

Diabetes mellitus is a chronic disease with a growing prevalence worldwide, however, the prevalence of its complications, including gastroenteropathy, is also increasing. The pathophysiology of diabetic gastroenteropathy (DH) combines hyperglycemia, vagus nerve dysfunction, decreased expression of nitric oxide synthase in the myenteric plexus, changes in the interstitial Cajal cell network, as well as oxidative stress. Clinical signs of DH are gastroesophageal reflux, gastroparesis, constipation, abdominal pain and diarrhea. Among the diagnostic methods are manometry with pH measurement (assessment of esophageal motility), gastric emptying scintigraphy, respiratory test (to assess gastroparesis), aspiration and cultivation of the contents of the jejunum (to diagnose bacterial overgrowth syndrome). To date, there is no definitive treatment for DH — an interdisciplinary approach is aimed at slowing the progression of the disease, relieving symptoms and restoring gastrointestinal function. Patients are recommended a diet low in simple sugars and high in fiber; optimization of glycemic control with a target glycemia of less than 180 mg/dl. As for drug therapy, the use of prokinetics and antiemetics is justified, and in case of excessive bacterial growth syndrome, antibacterial therapy (rifaximin) is carried out. Modern approaches to the treatment of DH are also accumulating, including the use of botulinum toxin, pyloroplasty and electrical stimulation of the stomach in individual patients. Despite the constant development of new treatments, they are not yet able to completely cure DH in the near future, which makes it necessary to conduct further research in this area.

## ВВЕДЕНИЕ

Диабетический гастропарез был впервые описан в 1945 г. Уэйном Ранделлсом, позже, в 1958 г., был введен термин gastroparesis diabeticorum [1]. Данная патология является одним из самых распространенных осложнений у пациентов с сахарным диабетом 1 и 2 типов (СД1 и СД2), она характеризуется ранней сытостью, длительной постпрандиальной полнотой, вздутием живота, тошнотой и рвотой, а также болью в животе [2]. Патофизиология диабетической гастроэнтеропатии (ДГ) сложна. Считается, что нейропатия энтеральной нервной системы, индуцированная гипергликемией, является одной из основных причин развития ДГ. Немаловажную роль играет уменьшение количества интерстициальных клеток Кахаля (ИКК) и кишечных глиальных клеток. С другой стороны, окислительный стресс и воспаление также влияют на регенеративные процессы в кишечнике и на его иннервацию [3]. Таким образом, ДГ является комплексным осложнением СД, обусловленным дисфункцией автономной нервной системы и дизрегуляцией секреции и действия гормонов, что проявляется нарушением моторики пищевода, желудка и кишечника и сопровождается характерными симптомами.

Несмотря на то что данное осложнение СД было описано пять десятилетий назад, его диагностика и лечение по-прежнему являются актуальной проблемой для врачей практического здравоохранения.

Настоящий обзор литературы сфокусирован на патофизиологических аспектах, клинической диагностике, а также привносит новые данные о лечении ДГ. Целью обзора является анализ литературы, посвященной патогенезу, современным методам диагностики и лечения ДГ.

Настоящий обзор литературы выполнен с целью критической оценки собранного материала. Произведен электронный поиск публикаций в базах данных PubMed, Science Direct Scopus и Google Scholar. Условиями поиска было наличие слов «diabetic gastroenteropathy», «gastroparesis», «diabetes mellitus», «сахарный диабет», «диабетическая гастроэнтеропатия» в заголовках, аннотациях и ключевых словах. Методологическую оценку исследований проводили в соответствии со стандартами PRISMA, включая оценку систематической ошибки. Авторы независимо друг от друга проанализировали те статьи, названия и аннотации которых были релевантны условиям поиска. Разногласия между авторами относительно приемлемости разрешали путем консенсуса. В обзор включены исследования, опубликованные за последние 10 лет. Анализу подвергали полные тексты статей и их аннотации.

## ЭПИДЕМИОЛОГИЯ

Общая численность пациентов с СД в Российской Федерации, состоящих на диспансерном учете, на 01.01.2021 г., по данным регистра, включающего 84 региона РФ, составила 4 799 552 (3,23% населения РФ), из них: СД1 — 5,5% (265,4 тыс.), СД2 — 92,5% (4,43 млн), другие типы СД — 2,0% (99,3 тыс.) [4].

Распространенность ДГ не установлена. В некоторых исследованиях упоминалась кумулятивная частота диабетического гастропареза за 10 лет, составившая 5,2% для пациентов с СД1 и 1% для пациентов с СД2 [5][6]. Однако из-за постоянного увеличения числа пациентов с СД2 распространенность ДГ среди них будет повышаться [7].

Что касается распространенности симптомов ДГ, то дисмоторика пищевода составляет 63%, рефлюкс — 41%, у 60% пациентов наблюдаются запоры, а у 20% — диарея [8]. Такие различия в распространенности, вероятно, связаны с отсутствием ранней диагностики симптомов ДГ.

## ФИЗИОЛОГИЯ

## Опорожнение желудка

Опорожнение желудка — это физиологический процесс, позволяющий осуществлять транзит переваренной пищи в двенадцатиперстную кишку [2]. Данный процесс является механическим и регулируется сложными нейрогуморальными механизмами [9][10]. Парасимпатическая регуляция осуществляется через блуждающий нерв, волокна которого проходят от ЖКТ к ядру одиночного тракта и затем через дорсальное ядро блуждающего нерва выходят в миентеральное сплетение. В стенке желудка формируются два двигательных контура: возбуждающий и тормозной, которые распределяются гетерогенным образом (допуская интегральную функцию в желудочной биомеханике) в теле желудка, антральном отделе, привратнике, а также в ИКК [9]. Кроме того, существует несколько нейротрансмиттеров, которые участвуют в нейрогуморальной регуляции на разных уровнях (ацетилхолин, норадреналин, ГАМК, дофамин и т.д.), а также гормонов, синтезируемых в поджелудочной железе, тонком кишечнике и нервной системе, некоторые из них задерживают опорожнение желудка (холецистокинин, лептин и глюкагоноподобный пептид-1), а другие — ускоряют (мотилин, грелин) [9].

Скорость опорожнения желудка разная в зависимости от консистенции и характера пищи, поэтому низкокалорийная и жидкая пища быстро покидает желудок [9–11]. Большая часть твердой пищи задерживается в желудке от 2 до 3 ч (т.к. она превращается в мягкие частицы с образованием кислотного химуса, после чего попадает в двенадцатиперстную кишку со средней скоростью 1–4 Ккал/мин) [12]. Однако опорожнение не всегда является полным, существует межпищевой период, в течение которого частицы пищи проходят без переваривания. В последнее время благодаря широкому развитию неинвазивных методов исследования, таких как сцинтиграфия, физиология опорожнения желудка стала более понятна [9]. Адекватная биомеханика опорожнения желудка является результатом нормальной координации между проксимальной и дистальной областями желудка [12]. Дно желудка служит резервуаром для его содержимого (в результате расслабления под действием оксида азота) [2][13], а затем действует как напорный насос. В теле желудка его содержимое перемешивается со скоростью 3 перистальтические волны в минуту [10][11][14], привратник действует как затвор, который пропускает частицы размером не более 1–2 мм [15]. В процессе опорожнения желудка определяются 2 фазы: дигестивная (транспортировка химуса в двенадцатиперстную кишку) и интердигестивная (полное опорожнение желудка от неперевариваемых частиц за счет перистальтических волн); гормоны, ускоряющие опорожнение, участвуют во второй фазе [9].

## ПАТОФИЗИОЛОГИЯ

Гастропарез определяется как замедленное опорожнение желудка при отсутствии механической обструкции. Хотя точный механизм желудочной дисфункции и происхождения симптомов неизвестен, существуют факторы, способствующие ее развитию, такие как гипергликемия, дисфункция блуждающего нерва, недостаточная экспрессия нейронной синтазы оксида азота (nNOS) в миентеральном сплетении, уменьшение количества и качества клеток-пейсмейкеров, собственных водителей ритма, ИКК, которые принимают участие в генерации перистальтических волн, а также окислительный стресс [15] (рис. 1). Моторные нарушения ЖКТ являются многофакторным процессом, в его основе лежат нарушения как центрального, так и периферического отделов вегетативной нервной системы, что проявляется нарушениями модулирующего воздействия вегетативной нервной системы в основном за счет превалирования тормозящей иннервации в сравнении с возбуждающей вследствие нарушения проведения нервных импульсов на уровне нервных волокон и сплетений [16].

**Figure:**
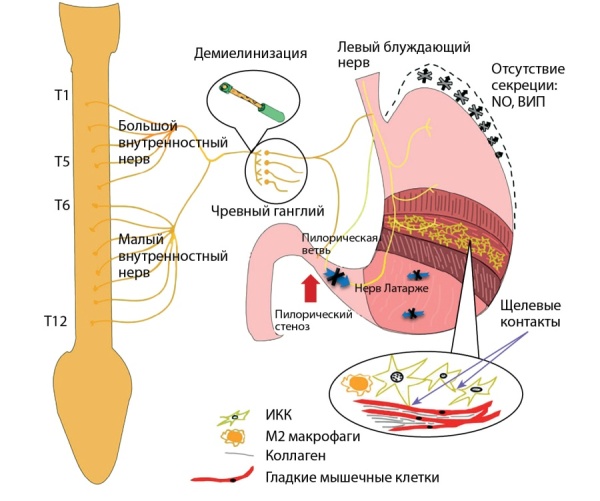
Рисунок 1. Патогенез диабетического гастропареза.Figure 1. Pathogenesis of diabetic gastroparesis.

Резкое повышение или снижение (вдвое) уровня глюкозы крови может вызывать соответственно задержку или ускорение опорожнения желудка [12][16]. Кроме того, нарушение опорожнения желудка может вызывать колебания гликемии, которые, в свою очередь, влияют на скорость опорожнения желудка, тем самым замыкая порочный круг. При наличии гипергликемического состояния возникают спазм привратника и гипомоторика в антральном отделе желудка, приводящая к задержке его опорожнения. Гипогликемическое состояние вызывает стимуляцию блуждающего нерва [15].

Аналогичным образом вагусная дисфункция играет роль в развитии диабетического гастропареза. Вагусная нейропатия приводит к снижению релаксации привратника, нарушению сокращения антрального отдела желудка, а также к нарушению антропилорической координации [17][18].

Изменения в энтеральной нервной системе также играют важную роль в патогенезе ДГ. Миентеральное сплетение составляет скопление нервных волокон, расположенных между продольными и круговыми мышцами кишечника, оно принимает участие в координации моторной функции желудка. Миентеральное сплетение состоит из возбуждающих (холинергических) и тормозных (нитрергических) двигательных нейронов, первичных афферентных нейронов и интернейронов [17]. Возбуждающие двигательные нейроны индуцируют мышечные сокращения, высвобождая нейротрансмиттеры, такие как ацетилхолин и субстанция P, в то время как тормозные нейроны расслабляют мышечную ткань путем высвобождения оксида азота, АТФ ивазоактивного интестинального пептида [13]. Патологические изменения в данных механизмах влияют на моторную регуляцию и приводят к задержке опорожнения желудка, нарушению аккомодации и желудочной дисритмии [15].

В экспериментах на NOD-мышах (Non-Obese Diabetes), так называемой модели диабета без ожирения, было выявлено обратимое снижение экспрессии желудочного nNOS, предполагается, что у пациентов с диабетом может существовать отрицательная регуляция nNOS без потери нитратных нейронов [13]. Исследование на крысах с диабетом, индуцированным введением стрептозотоцина (СТЗ), показало обратимую потерю nNOS после 4–8 нед, которая прогрессировала донеобратимой в результате апоптоза, вызванного окислительным стрессом после 12 нед. Поскольку активный фермент nNOS представляет собой димеризованный белок, потеря этой димеризации может вызывать нарушение нервно-мышечной функциив антральном отделе желудка крыс с СТЗ-диабетом [19].

Уменьшение количества ИКК было зарегистрировано в животных моделях, а также у пациентов с диабетическим гастропарезом. В моделях NOD-мышей и крыс с СТЗ-диабетом была обнаружена потеря ИКК как в теле желудка, так и в антральном отделе [15]. Консорциум клинических исследований гастропареза получил данные, отражающие корреляцию клеточных изменений в желудке (оценивали биопсийный материал) с симптомами пациентов, страдающих гастропарезом [20]. Авторы выявили связь между снижением ИКК и частотой опорожнения желудка. В отличие от данных, полученных в предыдущих исследованиях, у пациентов с СД экспрессия nNOS не была значительно снижена. Следует отметить, что биопсийный материал был собрану пациентов, перенесших установку желудочного нейростимулятора, и поэтому данная выборка не является репрезентативной для общей популяции пациентов с диабетическим гастропарезом [20].

Хорошо известно, что СД вызывает состояние окислительного стресса и способствует снижению азотной функции. Повышенный окислительный стресс в моделях мышей-NOD, обусловленный потерей макрофагальной гем-оксигеназы-1 (ГО-1), которая в норме защищает энтеральную нервную систему от воздействия свободных радикалов, был связан с уменьшением количества и качества ИКК, вызывающим задержку опорожнения желудка. Задержка опорожнения желудка связана с потерей макрофагальной ГО-1. Индукция ГО-1 обращает вспять задержку опорожнения желудка [2].

Немаловажную роль в патогенезе ДГ играют гуморальные воздействия, эффекты которых можно разделить на стимулирующие и тормозящие. Холецистокинин, гастрин и инсулин являются основными стимуляторами постпрандиальной моторики, причем известно, что инсулин воздействует опосредованно через центры блуждающих нервов. Гастрин обладает выборочной активацией моторики двенадцатиперстной кишки, а также верхних отделов тонкого кишечника [16].

Тормозящее влияние на моторную активность тонкой кишки обнаружено и у глюкагона. Грелин является гастроинтестинальным пептидом, который является стимулятором выработки гормона роста, а также отвечает за появление чувства голода. В эксперименте было показано, что грелин усиливает желудочную сократимость. Он вырабатывается «пустым» желудком и тонким кишечником, после чего поступает в кровь. Непосредственно перед приемом пищи уровень грелина в крови возрастает, и возникает чувство голода. Важная роль отводится глюкагоноподобному пептиду 1 и гастроинтестинальному полипептиду. Имеются данные, что глюкагоноподобный пептид 1 участвует в регуляции скорости опорожнения желудка, принимает непосредственное участие в глюкозозависимой регуляции постпрандиальной гликемии и обладает центральным механизмом модуляции чувства насыщения [16].

## КЛИНИЧЕСКИЕ ПРОЯВЛЕНИЯ

Распространенность желудочно-кишечной диспепсии выше у пациентов с СД, чем в общей популяции, примерно на 70% [21].

В результате нарушения механизмов моторики и секреции в ЖКТ диабетическая энтеропатия может поражать любой его участок, включая пищевод, поражения которого встречаются более чем у 60% больных СД [22][23]. Алгоритм диагностики ДГ представлен на рисунке 2.

**Figure:**
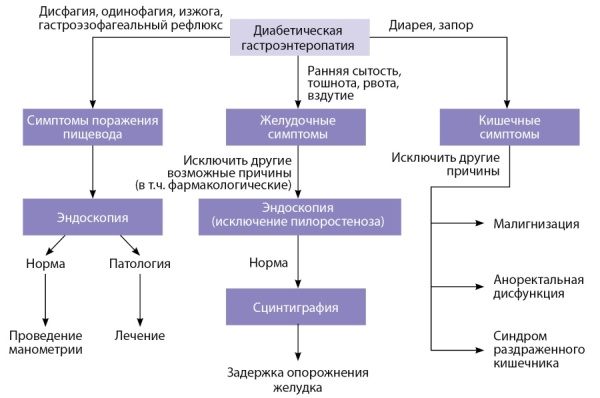
Рисунок 2. Алгоритм диагностики диабетической гастроэнтеропатии.Figure 2. Algorithm for the diagnosis of diabetic gastroenteropathy.

## Изменения пищевода

Нарушение моторики пищевода обычно сопровождается симптомами гастроэзофагеального рефлюкса или дисфагии. Тем не менее также может наблюдаться одинофагия, обычно связанная с кандидозом пищевода [22].

В некоторых случаях гастроэзофагеальный рефлюкс проявляется в виде кашля и ухудшения функции внешнего дыхания, усложняя диагностику [23]. Изжога связана с гастроэзофагеальным рефлюксом в 41% случаев [22].

Исследования у пациентов с дисфагией показали, что симптомы встречаются чаще у женщин и белых людей. Кроме того, около 45% больных СД с дисфагией имели моторные нарушения в пищеводе. При сравнении групп пациентов с дисфагией больные СД имели более высокий процент курения и индекс массы тела, чем группа пациентов с дисфагией без СД. Результаты манометрии показали, что пациенты, принимающие инсулин, имели более выраженную слабость глотания при отсутствии корреляции с уровнями гликозилированного гемоглобина [23]. Эпизоды острой гипергликемии были связаны с более низкой моторикой пищевода и большей дисфагией [10]. Кроме того, наличие вегетативной и периферической нейропатии иретинопатии связано с более высокой частотой эрозивного эзофагита по сравнению с пациентами без нейропатии [24].

## Желудочные расстройства

Клинические симптомы гастропареза включают тошноту, рвоту, раннее насыщение, постпрандиальную полноту, вздутие живота, отрыжку и дискомфорт в верхней части живота. Эти симптомы могут пересекаться с симптомами, наблюдаемыми при функциональной диспепсии [25][26].

Кардинальные симптомы гастропареза обычно присутствуют в комбинации, а не по отдельности. Хотя симптомы идиопатического и диабетического гастропареза схожи, рвота и раннее насыщение чаще встречаются при диабетическом гастропарезе, в то время как боль в животе чаще встречается при диабетическом гастропарезе [25].

Метаанализ 92 исследований показал, что задержка опорожнения желудка коррелирует с желудочно-кишечными симптомами, такими как тошнота (отношение шансов — ОШ 1,6; 95% доверительный интервал — ДИ 1,4–1,8), рвота (ОШ 2,0; 95% ДИ 1,6–2,7), раннее насыщение (ОШ 1,8; 95% ДИ 1,2–2,6) и незначительно с болями в животе (ОШ 1,5; 95% ДИ 1,0–2,2) при ОШ 2,0 [27]. У пациентов с СД наиболее сильная корреляция была обнаружена между ранним наступлением сытости и задержкой опорожнения желудка. Тем не менее нейропатия может приводить к изменениям моторики желудка и уменьшению выраженности симптомов.

У пациентов с СД1 было замечено, что женщины страдают гастропарезом чаще (5,8%, P<0,001), чем мужчины (3,5%, P<0,001), кроме того, гастропарез выявлялся у пациентов преимущественно старшей возрастной группы. Пациенты с гастропарезом обычно имели длительную продолжительность диабета, более высокие уровни гликозилированного гемоглобина, а также более частые эпизоды тяжелой гипогликемии [28], аналогичное влияние пола также наблюдалось у пациентов с СД2. Причина различий в распространенности диабетического гастропареза между мужчинами и женщинами неизвестна; однако моторика желудка зависит от нейронального синтеза оксида азота, нарушение димеризации nNOS вызывает снижение синтеза NO, что приводит к снижению релаксации гладкой мускулатуры желудка.

Среди факторов риска ожирение у пациентов с СД повышает вероятность развития гастропареза в 10 раз. Имеются данные, что 50% пациентов с идиопатическим гастропарезом имеют избыточный вес или страдают ожирением, при этом выраженность симптомов зависит от индекса массы тела. Пациенты с ожирением в меньшей степени страдают от потери аппетита, но имеют более высокую частоту развития гастроэзофагеального рефлюкса [10].

## Расстройства кишечника

Наиболее распространенными симптомами со стороны кишечника являются запор, диарея, боль и вздутие живота. Частота хронических запоров выше (25%), чем хронической диареи (5%), в группе пациентов с СД [25].

Исследование NHANES (National Health and Nutrition Examination Survey) показало, что 25% пациентов с СД имели желудочно-кишечные симптомы, но, в отличие от предыдущих исследований, в этом сообщалось, что только хроническая диарея была более распространена у пациентов СД, чем в группе пациентов, не страдающих СД (11,2% против 6,0%, P<0,0001). Однако в распространенности хронических запоров подобных различий выявлено не было. Кроме того, пациенты с СД и хронической диареей, как правило, принимали больше гипогликемических препаратов, чаще всего метформин; диабетики с хроническими запорами имели сниженную функцию почек. Не было обнаружено значимой связи между кишечными симптомами и наличием нейропатии, уровнями гликозилированного гемоглобина и продолжительностью СД [29].

Диарея обычно длится более 6 нед, водянистая, безболезненная, без примеси крови, и ее связь с продолжительностью СД является вариабельной. Хроническая диарея чаще встречается у женщин, развивается примерно через 8 лет после постановки диагноза СД на фоне нормального стула или даже запора и проявляется резким увеличением частоты и объема стула. Ночная диарея и недержание кала являются двумя наиболее отличительными признаками диабетической диареи [3].

## Заболевания прямой кишки и анального отверстия

Недержание кала чаще встречается у больных СД, это связано с продолжительностью заболевания и наличием микрососудистых осложнений. У больных СД снижается тонус внутреннего анального сфинктера [25].

Распространенность недержания кала колеблется в пределах 7–15%, однако пациенты не всегда говорят об этом симптоме. Кишечные расстройства, такие как диарея, являются независимым фактором риска недержания кала. Кроме того, курение, ожирение, пожилой возраст, малоподвижный образ жизни и женский пол также являются факторами риска [30].

## ШКАЛЫ ОЦЕНКИ СИМПТОМОВ

В связи с необходимостью контроля желудочно-кишечной симптоматики у пациентов с СД предпочтение отдается анкетированию. В последние годы эти вопросники меняются, но, несмотря на это, многие исследователи продолжают использовать непроверенные инструменты для оценки желудочно-кишечных симптомов. Хотя анкетирование является стандартом для симптоматической оценки, оно может быть не оптимальным для мониторинга изменений симптомов с течением времени ввиду наличия систематических ошибок при повторных опросах [26].

В анкетах должны использоваться четкие формулировки, оцениваться все соответствующие симптомы, а также они должны давать сопоставимые результаты при оценке пациентов с постоянными симптомами и выявлять клинически значимые изменения [31].

Наиболее широко используемыми опросниками являются индекс тяжести желудочно-кишечных симптомов (PAGI-SYM), индекс кардинальных симптомов гастропареза (GCSI), шкала оценки желудочно-кишечных симптомов (GSRS), анкета для оценки запора (PAC-SYM), а также анкета для оценки качества жизни при запорах (PAC-QOL) [26].

PAGI-SYM оценивает выраженность симптомов со стороны верхних отделов ЖКТ. Он содержит 20 пунктов и разделен на 6 разделов: изжога-регургитация, постпрандиальная полнота-раннее насыщение, тошнота-рвота, вздутие живота, боль вверхней и нижней частях живота. Данный индекс полезен для оценки гастроэзофагеального рефлюкса, диспепсии и гастропареза [22][26]. GCSI включает в себя три шкалы, которые оценивают тошноту-рвоту, постпрандиальную полноту-раннее насыщение и вздутие живота. GCSI в течение 2 нед позволяет оценить тяжесть симптомов гастропареза. Для оценки эффективности лечения был разработан ежедневный дневник индекса кардинальных симптомов гастропареза (ANMS GCSI-DD) [32].

GSRS содержит 15 пунктов, объединенных в 5 групп: рефлюкс, боль в животе, расстройства желудка, диарея и запор. GSRS обеспечивает более широкую перспективу, чем предыдущий вопросник; тем не менее, по-прежнему необходимы дополнительные исследования для адекватной корреляции между баллами анкеты и объективными показателями желудочно-кишечных расстройств. Тем не менее этот инструмент не очень чувствителен к гастроэзофагеальному рефлюксу [21][32].

PAC-SYM и PAC-QOL были разработаны для оценки тяжести симптомов и качества жизни у пациентов с запорами. Первая анкета состоит из 12 пунктов и трех групп: абдоминальные симптомы, ректальные симптомы и характеристика стула. Опросник M-PAC-SYM был разработан для пациентов с хроническими запорами и может быть полезен для оценки функциональных запоров, связанных с СД [33].

## ОСОБЕННОСТИ ДИАГНОСТИКИ

## Заболевания пищевода

Выявление симптомов со стороны пищевода у больных СД проводится так же, как и у пациентов без диабета. Типичных симптомов гастроэзофагеального рефлюкса бывает достаточно для постановки диагноза. Эндоскопия не рекомендуется в диагностических целях, однако она может быть эффективна для диагностики осложнений в слизистой оболочке пищевода, а также для выявления кандидозного эзофагита [21][29].

Нарушение моторики пищевода можно оценить с помощью манометрии и видеофлюороскопического исследования глотания. Измерение pH может быть полезно при оценке гастроэзофагеального рефлюкса с использованием мониторинга импеданса, что позволяет оценить транзит воздуха и жидкости [21].

Манометрия позволяет оценить время транзита пищи в пищеводе, а также давление в нижнем пищеводном сфинктере. Манометрия в сочетании с измерением pH служит лучшим способом оценки моторики пищевода [21][23].

## Желудочные расстройства

Сцинтиграфия опорожнения желудка является методом выбора для диагностики гастропареза. При использовании данного метода пациент принимает пищу, меченную технецием-99, после чего оценивается опорожнение желудка. Также его можно использовать для измерения времени прохождения пищи в тонком и толстом кишечнике. Показания к проведению сцинтиграфии включают инсулинозависимый СД с наличием постпрандиальных симптомов или СД с плохим гликемическим контролем, неязвенную диспепсию, тяжелый эзофагит, вызванный рефлюксом, тошноту, рвоту, потерю веса, дискомфорт в верхней части живота, раннее наступление сытости, а также может использоваться для оценки ответа на лечение прокинетическими препаратами. После приема меченой пищи жидкое содержимое быстро проходит через желудок, в то время как твердые компоненты пищи концентрируются в дне желудка [34]. В норме остаточное желудочное содержимое должно составлять менее 60% через 2 ч после приема пищи и менее 10% через 4 ч. Более высокие значения указывают на наличие гастропареза. Задержка более 60% желудочного содержимого через 2 ч имеет чувствительность 100% и специфичность 20%. Задержка более 10% желудочного содержимого через 4 ч имеет чувствительность 100% и специфичность 70% для диагностики гастропареза [35].

При проведении дыхательного теста нерадиоактивный изотоп C13 связывается с легкоусвояемым веществом, как правило, октановой кислотой, которая смешивается с твердой пищей, всасывается в проксимальном отделе тонкой кишки с последующим метаболизмом в печени до C13–C02, который можно измерить в выдыхаемом воздухе. Дыхательный тест имеет чувствительность 89% и специфичность 80%. По сравнению со сцинтиграфией дыхательный тест проще в выполнении и не имеет радиоактивной нагрузки на организм, однако сопутствующие заболевания, такие как целиакия, могут влиять на диагностическую значимость данного теста [26]. Дыхательный тест проводится в течение 4 ч после 8-часового голодания. Образцы выдоха собирают перед едой и затем каждые 30 мин [36].

Капсульная эндоскопия позволяет непрерывно измерять давление, pH и температуру при движении по ЖКТ. Процедура включает в себя стандартизированное питание с последующим приемом капсулы. Данные из капсулы передаются на приемный блок. С помощью этого метода было установлено, что около 44% пациентов с СД1 и разной степени сенсомоторной нейропатией имели нарушение моторики в одном или нескольких сегментах ЖКТ независимо от наличия симптомов [37]. Изменения pH в различных сегментах ЖКТ могут отражать ферментацию в области слепой кишки, которая оказывает влияние на время транзита содержимого в толстой кишке. Капсульная эндоскопия имеет существенные ограничения, обусловленные высокой стоимостью процедуры и необходимостью ее проведения в специализированных центрах. Только 52,5% результатов согласуются со сцинтиграфией, поэтому у пациентов с подозрением на гастропарез требуются дополнительные исследования [26][36].

Также возможно использование рентгеноконтрастных методов исследования, которые имеют широкую доступность и пользу для выявления значительных изменений во времени прохождения содержимого по кишечнику и опорожнения желудка. Однако эти методики не обладают высокой чувствительностью и специфичностью для диагностики гастропареза, поэтому необходимо проведение дополнительных исследований [26].

Все вышеупомянутые методы исследования могут выполняться только после исключения механической обструкции, которая может вызывать симптомы, подобные гастропарезу [36].

## Кишечные расстройства

Одной из фундаментальных причин желудочно-кишечных симптомов является избыточный бактериальный рост в кишечнике, диагностическими стандартами этого состояния служат аспирация и культивирование содержимого тощей кишки. С целью аспирации необходимо прибегать к эндоскопии, в результате чего возникает высокая вероятность внешнего бактериального обсеменения и получения ложноотрицательных данных. Дыхательные тесты не показали адекватной чувствительности [21][26][36].

При запорах возможно использование аноректальной манометрии для оценки расстройства дефекации. Оценка времени прохождения содержимого по кишечнику также возможна с помощью вышеописанных методов исследования. Важно проводить тщательное исследование кишечника, чтобы исключить злокачественные новообразования [23].

Лекарства, используемые пациентами, следует рассматривать как возможную причину диареи и запоров. Кроме того, из-за более высокой распространенности целиакии у пациентов с диабетом, чем среди населения в целом, оправданным является скрининг с использованием серологических тестов [21][23].

## ЛЕЧЕНИЕ

На сегодняшний день отсутствуют лекарства от ДГ. Поэтому основными целями лечения являются задержка прогрессирования заболевания, устранение симптомов, предупреждение осложнений, а также восстановление нормальной функции. Лечение ДГ является мультидисциплинарным. Оно требует участия нескольких специалистов, таких как гастроэнтеролог, эндокринолог, диетолог, психолог, интервенционный радиолог и хирург [21].

Дидактическим путем мы разделили лечение гастропареза и диабетической энтеропатии, учитывая, что обе формы заболевания могут сосуществовать (рис. 3).

**Figure:**
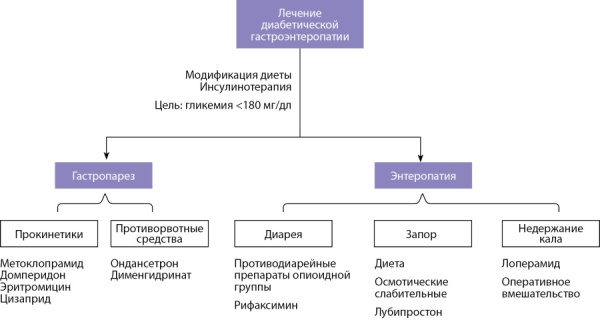
Рисунок 3. Лечение диабетической гастроэнтеропатии.Figure 3. Treatment of diabetic gastroenteropathy.

## Лечение диабетического гастропареза

Большинство пациентов, как правило, имеют легкое и умеренное течение заболевания и, следовательно, хорошо реагируют на диетические модификации и гликемический контроль, а также на прокинетические и противорвотные препараты [38][39]. Небольшой процент пациентов имеют тяжелое течение заболевания, характеризующееся плохой оральной толерантностью, хроническим недоеданием, а также частыми госпитализациями [3].

С другой стороны, также важно контролировать сопутствующие заболевания, которые обычно имеются у пациентов с диабетическим гастропарезом, такие как гастроэзофагеальный рефлюкс, дисмоторика кишечника, дефицит витамина D и других микроэлементов, бактериальные и грибковые инфекции ЖКТ, а также макрососудисто-микрососудистые осложнения СД [40][41].

## Рекомендации в отношении питания

Модификация диеты представляет собой первую линию в лечении гастропареза, например, интервальное питание уменьшает выраженность симптомов со стороны верхних отделов ЖКТ, хотя его эффективность не была четко установлена [42][43].

Пациенты с диабетическим гастропарезом, как правило, потребляют более низкое количество калорий, чем рекомендуется, а также испытывают значительный дефицит микро- и макроэлементов [44]. Диета, основанная на низком содержании простых сахаров и продуктов, богатых клетчаткой, предложенная Американской диабетической ассоциацией (АДА) [45], обычно не имеет пользы для пациентов с диабетическим гастропарезом [3].

Рекомендуются меры, способствующие опорожнению желудка или не задерживающие его [3]. Именно поэтому потребление жиров, клетчатки и газированных жидкостей должно быть сведено к минимуму [36]. Рекомендуется использовать поэтапный подход, начиная с жидкой пищи, обладающей высокой питательной ценностью (супы, коктейли), а затем добавлять твердую пищу, которая не задерживает опорожнение желудка [3]. Продукты с низким содержанием жира должны употребляться 4–5 раз вдень. Пациентов необходимо проинструктировать о том, что во время приема пищи необходимо запивать ее жидкостью, а также сидеть или ходить на протяжении 1–2 ч после еды.

Если все эти рекомендации неэффективны, пациенту следует предложить потреблять общее количество калорий в жидкостях (супы или коктейли), т.к. способность к опорожнению жидкого содержимого желудка часто сохраняется [41].

Необходимо отметить важность просвещения пациентов и их семей по поводу диетических модификаций. Установлено, что распространенность консультаций по питанию у больных СД с гастропарезом составляет менее 40% [44].

## Гликемический контроль

Жизненно важно обеспечить адекватный гликемический контроль, чтобы свести к минимуму острые проявления гастропареза и улучшить опорожнение желудка [3][43].

Лечение, используемое для достижения адекватного контроля гликемии, должно быть индивидуализировано [3][43]. Пероральные сахароснижающие средства не рекомендуются к применению у пациентов с СД2 при наличии клинически значимого диабетического гастропареза. На фармакокинетику этих препаратов оказывает влияние задержка опорожнения желудка; поэтому данные препараты не являются идеальными для обеспечения эффективного гликемического контроля [3][43].

Аналогичным образом неблагоприятные эффекты противодиабетических препаратов также играют важную роль, например побочные реакции со стороны ЖКТ на фоне применения метформина; гипогликемия, обусловленная препаратами сульфонилмочевины; диарея и вздутие живота при использовании ингибиторов альфа-глюкозидазы; противоречивый эффект на опорожнение желудка оказывают ингибиторы дипептидилпептидазы-4, а также ингибиторы натрий-глюкозного ко-транспортера-2. Относительно инъекционной терапии агонисты глюкагоноподобного пептида-1 могут усугублять симптомы гастропареза [3][43].

Пациентам с диабетическим гастропарезом и СД1, а также большинству пациентов с СД2 потребуется инсулин для обеспечения гликемического контроля [46]. Рекомендуется использовать прандиальный инсулин после еды, чтобы предотвратить постпрандиальную гипогликемию, т.к. полноценный прием пищи может быть затруднен. Многократный прием небольших порций еды, активный мониторинг глюкозы и частое использование небольших доз инсулина быстрого действия рекомендуются для предотвращения постпрандиальной гипергликемии [3]. В настоящее время рекомендуемым методом обеспечения гликемического контроля у пациентов с диабетическим гастропарезом является непрерывная подкожная инфузия инсулина (инсулиновая помпа), которая показала свою эффективность в оптимизации гликемии, а также снижении частоты госпитализаций [47]; однако нельзя забывать об экономических издержках, которые влечет за собой данный метод лечения. Применение предварительно смешанных инсулинов не рекомендуется в этой группе пациентов [7].

## Фармакологическое лечение

Препараты, наиболее часто используемые при лечении диабетического гастропареза, обычно включают прокинетические и противорвотные средства [3].

Метоклопрамид является антагонистом D-2 рецепторов, обладающим противорвотным и прокинетическим эффектами, он усиливает сокращения антрального отдела желудка, а также координирует антральную и дуоденальную моторику. Максимальная суточная доза составляет 40 мг/сут. Его можно использовать парентерально при наличии выраженных симптомов. Среди побочных эффектов отмечается повышение концентрации пролактина в сыворотке крови. Гинекомастия игалакторея могут возникать у взрослых мужчин, подростков и детей, в то время как у взрослых женщин может развиваться олигоменорея. Кроме того, он может стимулировать синтез альдостерона и вызывать неконтролируемую гипертензию у пациентовс первичным гиперальдостеронизмом, а также удлинять интервал Q–T у некоторых пациентов [3]. Это единственный препарат, одобренный Управлением по санитарному надзору за качеством пищевых продуктов и медикаментов США (FDA) для лечения гастропареза; однако в феврале 2009 г. FDA и Европейское агентство по лекарственным средствам установили ограничение для длительного использования метоклопрамида (более 12 нед) из-за риска развития необратимой поздней дискинезии, что ограничило его применение [39].

Домперидон также является антагонистом рецепторов D-2, имеет одинаковую эффективность с метоклопрамидом, но с меньшим побочным воздействием на центральную нервную систему, поскольку он не проникает через гематоэнцефалический барьер [48]. Было установлено, что суточная доза 10–30 мг, вводимая за 30 мин до еды и перед сном, уменьшала выраженность симптомов со стороны ЖКТ, а также количество госпитализаций из-за гастропареза.

Эритромицин является макролидом с агонистическим действием на мотилиновые рецепторы в ЖКТ. Он увеличивает опорожнение желудка дозозависимым образом. Было показано, что эритромицин стимулирует опорожнение желудка у пациентов сгастропарезом. Суточная доза 50–100 мг, вводимая 3 раза в сутки, в сочетании с модификацией диеты может быть эффективна для контроля гастропареза. Также были выявлены случаи удлинения интервала Q–T [3][9].

Цизаприд является мощным прокинетическим средством, которое, действуя в желудке через 5-гидрокситриптаминовые рецепторы, ускоряет опорожнение желудка от твердой пищи и снижает выраженность диспепсических симптомов [3].

Также существуют прокинетические препараты, которые находятся на этапе разработки, среди которых агонисты мотилина, агонисты грелина и новые агонисты 5-гидрокситриптаминовых рецепторов 4 типа [3][9].

Как тошнота, так и рвота являются наиболее инвалидизирующими симптомами у пациентов с диабетическим гастропарезом. В качестве противорвотных средств можно использовать агонисты серотонина, например, ондансетрон в дозе 4–8 мг 2 раза в сутки, или антагонисты гистаминовых рецепторов 1 типа, например, дименгидринат в дозе 50 мг 4 раза в сутки. Оба класса препаратов часто используются отдельно или в комбинации с прокинетическими средствами [3].

## Другие методы лечения

Ботулинический токсин, мощный ингибитор нервно-мышечной передачи, был постулирован для улучшения опорожнения желудка и уменьшения выраженности симптомов гастропареза за несколько месяцев; однако двойные слепые рандомизированные исследования показали, что улучшение опорожнения желудка при его использовании не приводит к коррекции симптомов [49].

Аналогичным образом существует интерес к роли привратника в развитии гастропареза, исходя из этого, некоторые авторы предлагают пероральную эндоскопическую миотомию желудка (G-POEM) в качестве метода лечения у рефрактерных пациентов. P. Mekaroonkamol и соавт. описали 3 случая пациентов с рефрактерным гастропарезом различной этиологии, которые излечились после проведения G-POEM [50].

Для группы пациентов с тяжелым течением гастропареза и рефрактерностью к терапии может быть эффективна электрическая стимуляция желудка. Было показано, что она уменьшает выраженность тошноты и рвоты, а также улучшает общее состояние и качество жизни пациентов после 6 мес терапии [51][52].

Значительное число пациентов нуждаются в хирургическом лечении ввиду рефрактерности к другим вышеописанным методам терапии. Основная роль хирургического лечения заключается в облегчении симптомов, декомпрессии желудка, обеспечении доступа к энтеральному питанию, а также стимуляции опорожнения желудка [3].

## Лечение диабетической энтеропатии

Поражение кишечника при СД имеет различные проявления: хроническая диарея (вовлечение тонкой кишки), запор при поражении толстого отдела кишечника, а также недержание кала. Все они объясняются мультисистемной природой заболевания (вегетативная нейропатия, инфекционное поражение, аутоиммунный характер поражения при СД 1 типа) [39][50][53].

## Хроническая диарея

Распространенность хронической диареи у больных СД колеблется от 3,7 до 22% [53]; по сравнению с общей популяцией, у них примерно в 2 раза выше риск развития диареи [31]. Важной частью лечения является оценка статуса гидратации и баланса электролитов, на которые предстоит воздействовать. Как и при гастропарезе, целью лечения является достижение адекватного гликемического контроля и диетической модификации. Если эти первоначальные меры оказываются неэффективными, лекарственная терапия противодиарейными препаратами опиоидной группы должна назначаться с осторожностью из-за их возможной токсичности: мегаколон и риск развития синдрома избыточного бактериального роста [10].

При развитии синдрома избыточного бактериального роста в тонком кишечнике следует начать антибактериальную терапию. Рифаксимин является лучшим научно обоснованным средством для лечения данной патологии, он обладает местным действием в ЖКТ и уменьшает выраженность симптомов у 33–99% пациентов [54]. Аналоги соматостатина, такие как октреотид и ланреотид, также улучшают состояние пациентов [8]. Другой распространенной причиной диареи у больных СД является прием метформина, который снижает всасывание желчных солей и искусственных подсластителей в подвздошной кишке, он расщепляется кишечной флорой с образованием водорода и короткоцепочечных жирных кислот, которые являются причиной развития диареи [55].

Тем не менее специфического лечения хронической диареи у больных СД не существует. Применение аналогов соматостатина показано при неэффективности других методов лечения.

## Запор

Когда запор является основной жалобой, хорошая гидратация, питание с высоким содержанием клетчатки и обычные физические нагрузки являются первой линией терапии. Рандомизированные плацебо-контролируемые клинические исследования показывают, что прием натурального подорожника (10 г два раза в день) или льняного семени (10 г два раза в день) уменьшает симптомы запора и улучшает гликемический контроль у пациентов с СД2. На сегодняшний день отсутствуют исследования, оценивающие эффекты слабительных средств у пациентов с запорами на фоне ДГ [56][57]. Основное лечение фокусируется на устранении симптомов с использованием диеты, способствующей размягчению стула, а также слабительных средств.

Хотя и без особых убедительных доказательств эффективности, можно предложить использовать осмотические слабительные средства, например полиэтиленгликоль; при недостаточной эффективности могут быть добавлены стимулирующие слабительные, такие как бисакодил и пикосульфат. Лубипростон, активатор хлоридных каналов, увеличивает секрецию толстой кишки, уменьшая время прохождения содержимого по кишечнику и повышая количество спонтанных дефекаций у пациентов с запорами на фоне СД [47][58].

## Недержание кала

При лечении недержания кала, которое часто усугубляется диареей, приоритетом является выявление основной причины диареи и ее устранение. Часто состояние улучшается само по себе на фоне адекватного гликемического контроля [8]. Лечение рефрактерных случаев представляет определенные сложности и иногда требует наложения стомы [47][50].

Нейромодулирующая электростимуляция крестцового нерва является новой методикой лечения недержания кала и восстановления чувствительности в анальном канале. Данный метод лечения является перспективным, однако требуется проведение дополнительных исследований у пациентов с ДГ [59].

## ЗАКЛЮЧЕНИЕ

СД является хроническим заболеванием с растущей распространенностью во всем мире, вместе с тем увеличивается и распространенность его осложнений, включая гастроэнтеропатию. Патофизиология диабетической гастроэнтеропатии объединяет гипергликемию, дисфункцию блуждающего нерва, снижение экспрессии синтазы оксида азота в миентеральном сплетении, изменения в интерстициальной клеточной сети Кахаля, а также окислительный стресс.

Клиническими признаками ДГ являются гастроэзофагеальный рефлюкс, гастропарез, запор, боль в животе и диарея. Среди методов диагностики выделяют манометрию с измерением pH (оценку моторики пищевода), сцинтиграфию опорожнения желудка, дыхательный тест (с целью оценки гастропареза), аспирацию и культивирование содержимого тощей кишки (с целью диагностики синдрома избыточного бактериального роста). На сегодняшний день не существует окончательного лечения ДГ — междисциплинарный подход направлен на замедление прогрессирования заболевания, облегчение симптомов и восстановление функции ЖКТ. Пациентам рекомендуются диета с низким содержанием простых сахаров и высоким содержанием клетчатки, оптимизация гликемического контроля при целевой гликемии менее 180 мг/дл. Что касается медикаментозной терапии, оправдано использование прокинетиков и противорвотных средств, а при возникновении синдрома избыточного бактериального роста — проведение антибактериальной терапии (рифаксимин).

Также накапливаются современные подходы к лечению ДГ, включая использование ботулинического токсина, пилоропластики и электрической стимуляции желудка у отдельных пациентов. Несмотря на постоянную разработку новых методов лечения, они пока не способны полностью излечить ДГ в ближайшем будущем, что делает необходимым проведение дальнейших исследований в данной области.

## ДОПОЛНИТЕЛЬНАЯ ИНФОРМАЦИЯ

Источники финансирования. Работа выполнена по инициативе авторов без привлечения финансирования.

Конфликт интересов. Авторы декларируют отсутствие явных и потенциальных конфликтов интересов, связанных с содержанием настоящей статьи.

Участие авторов. Кузнецов К.О. — разработка концепции и дизайна исследования, получение и анализ данных, интерпретация результатов; Михеева А.Ю. — разработка дизайна исследования, написание статьи; Ишмухаметова А.А. — анализ данных, написание статьи; Толстых Т.А. — интерпретация результатов, написание статьи; Галляметдинова А.Р. — получение и анализ данных, редактирование статьи; Ботирова З.У. — интерпретация результатов, редактирование статьи; Забирова А.А. — анализ данных, редактирование статьи; Шарипова А.Ш. — получение данных, редактирование статьи; Шайхлисламова А.Б. — получение данных, редактирование статьи; Абдрахманова Д.Р. — получение данных, редактирование статьи. Все авторы внесли равный вклад в написание статьи и одобрили ее финальную версию перед публикацией, выразили согласие нести ответственность за все аспекты работы, подразумевающую надлежащее изучение и решение вопросов, связанных с точностью или добросовестностью любой части работы.
